# Clinical characteristics and outcomes of non-obese patients with idiopathic intracranial hypertension: a retrospective cohort study

**DOI:** 10.1007/s10072-026-09182-2

**Published:** 2026-06-19

**Authors:** Zhonghua Ma, Hanqiu Jiang, Shilei Cui, Jingting Peng, Jiawei Wang

**Affiliations:** 1 Department of Neurology, Beijing Tongren Hospital, Capital Medical University, Beijing, China; 2 Department of Neurology, Beijing Tongren Hospital, Xicheng District, Beijing, China

**Keywords:** Idiopathic intracranial hypertension, Non-obese, Risk factors, Neuroimaging

## Abstract

**Background:**

Idiopathic intracranial hypertension (IIH) in non-obese patients represents a notable subtype of the condition, and its underlying causes require further investigation. This study aimed to analyse the clinical features and risk factors associated with non-obese IIH patients.

**Methods:**

A retrospective analysis was conducted on 252 adult patients with clinically confirmed IIH between January 2015 and December 2023. Data on epidemiological characteristics, clinical manifestations, and imaging findings were reviewed. The cases were classified into two groups: obese group (body mass index, BMI≥28 kg/m²) and non-obese group(BMI<28 kg/m²). A comparative assessment of clinical data was performed between the groups, including demographic information, clinical findings, medical history, laboratory results, radiological indices, diagnosis, treatment, and prognosis.

**Results:**

A total of 96 obese IIH and 82 non-obese IIH cases were included in this observation. Compared to the obese IIH group, non-obese cases were much older (37.94 ± 17.05vs.34.15 ± 8.74 year-old, *P* = 0.044), exhibited a lower frequency of abnormal radiological signs indicating intracranial hypertension (58.54%vs72.92%; *P* = 0.043), more severe visual impairment, and a reduced remission rate (60.98% vs. 81.25%%, *P* = 0.003). Additionally, 71 patients (86.59%) in the non-obese group had identifiable risk factors for IIH, including over-weight (63cases), obstructive sleep apnoea syndrome (OSAS) (36 cases), and weight gain (12cases). The mean duration of final follow-up was 15.02 ± 2.82 months. Univariate analysis showed that BMI, disease duration before treatment, visual field grading, and non-obese were significantly associated with poor best corrected visual acuity(BCVA) outcome (*P*<0.05). BCVA before treatment and imaging findings showed marginally significant associations (*P*<0.1). Multivariate logistic regression revealed that non-obese group (OR = 9.16, 95%CI: 1.09–77.18, *P* = 0.041) and longer disease duration (OR = 1.14, 95%CI: 1.05–1.24, *P* = 0.002) were independent risk factors for poor BCVA outcome. Poor BCVA before treatment showed a marginally significant trend toward poor prognosis (*P* = 0.089).

**Conclusions:**

Non-obese patients with IIH tend to exhibit more severe visual impairment and longer disease duration. Close monitoring and early intervention are recommended for non-obese patients with a long disease course.

## Background

IIH is a rare disorder characterised by elevated intracranial pressure(ICP) of unknown origin. It typically manifests with symptoms such as headaches, blurred vision, and papilloedema. Although the precise pathogenesis remains incompletely understood, IIH predominantly affects women and is strongly associated with obesity [1]. Population-based studies have shown that the incidence of IIH is low in Asia compared to that of the United Sates (US) or Europe: 0.9–1.07 per 100,000 per year in the US, 0.28–2.36 in Europe, and 0.03 in Japan [2]. A typical IIH refers to cases occurring in childbearing-aged female with a body mass index (BMI) above 30 kg/m² [3]. The clinical presentation of IIH in non-obese patients(BMI<30 kg/m²) may differ from that of typical IIH, which suggests possible differences in underlying pathophysiological mechanisms [4]. Consequently, a comprehensive diagnostic evaluation is necessary to exclude secondary causes of elevated intracranial pressure. This should include a detailed medical history, laboratory investigations, and extensive neuroimaging [5, 6]. Without timely diagnosis and appropriate management, IIH can lead to significant visual impairment, adversely affecting patients’ quality of life and functional capacity. This study aimed to analyse and summarise the clinical characteristics and risk factors associated with non-obese IIH.

## Methods

### Patients

This retrospective study analysed 252 adult patients with a presumed diagnosis of IIH, who were managed within the Department of Neurology at Beijing Tongren Hospital, Capital Medical University, Beijing, China, between January 2015 and December 2023. The study was approved by the Institutional Review Board of our hospital (TRECKY2018-052), and informed consent was obtained from all participants either via written informed consent signed at the time of clinical visit, or electronic consent by e-mail or message for those without pre-existing written documentation. Demographic information, including age, sex, height, and weight, was collected and analysed. A standardised protocol was employed for the review of medical records. Clinical data pertaining to signs and symptoms, ophthalmic and neurological examinations, neuroimaging results, medical history, laboratory investigations, treatment methods, follow-up information and visual outcomes were reviewed.

The inclusion criteria for IIH consisted of: (1) diagnosis of IIH based on the criteria defined by Friedman et al. [7]; (2) absence of alternative and more plausible causes of optic neuropathy.

Exclusion criteria applied to all participants included: (1) history of optic neuropathy; (2) presence of central nervous system (CNS) masses or other primary causes of elevated intracranial pressure; (3) treatment with topiramate or acetazolamide for more than two weeks before diagnosis of IIH; (4) failure to fulfil the diagnostic criteria for IIH; (5) coexisting neurological comorbidities; and (6) incomplete neuroimaging, clinical documentation, or lack of informed consent.

Prior to inclusion in the study registry, all patient cases were re-evaluated independently by two authors and a radiologist to confirm eligibility. A retrospective review of clinical data was conducted at two time points: before treatment and final follow-up.

Body weight classification was based on the Chinese adult BMI(kg/m^2^) criteria, whereby participants were categorised as follows: normal weight (18.5 ≤ BMI < 24), overweight (24 ≤ BMI < 28), or obese (BMI ≥ 28). A BCVA worse than 0.1 was deemed poor visual outcome in this study.

Visual impairment was categorised as severe (BCVA < 0.1), moderate (BCVA0.1-0.4), or mild (BCVA ≥ 0.5). Severity of papilloedema was graded according to the modified Frisén scale (MFS) [8, 9], which ranges from 0 (absent) to 5 (severe). Two neuro-ophthalmologists independently assessed all fundus images. Any discrepancies in grading were resolved through consensus.Visual field (VF) results were independently evaluated by two neuro-ophthalmologists according to the MD-based grading scale proposed by Shah et al. [10], as follows: Grade 0, normal visual field; Grade 1, MD < 4.0 with presence of a visual field defect; Grade 2, MD 4.0–11.9; Grade 3, MD 12.0–19.9; and Grade 4, MD > 20.0.

### Statistical analysis

Statistical analyses were performed using SPSS version 24.0. Categorical data are presented as numbers and percentages, while continuous variables are expressed as mean ± standard deviation. Comparisons of continuous data were conducted using the independent samples t-test or the Mann–Whitney U test, as appropriate. Categorical variables were compared using Pearson’s chi-square test or Fisher’s exact test. A two-tailed p-value of less than 0.05 was considered statistically significant. Univariate and multivariate logistic regression analyses were conducted to identify independent factors associated with visual function recovery. Results were expressed as odds ratios (OR) with 95% confidence intervals (95% CI).

## Results

**Fig. 1:**
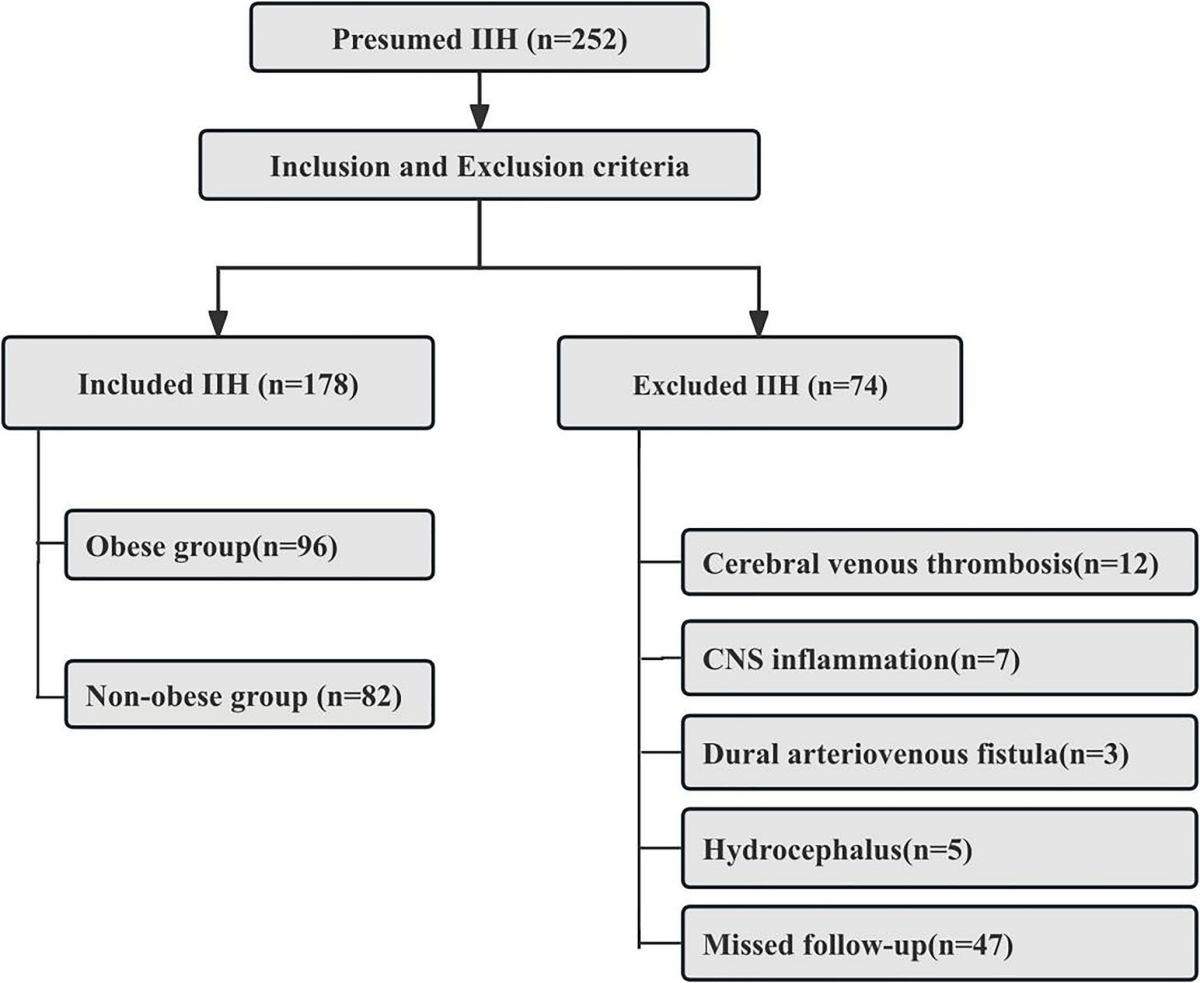
Flow chart of enrollment

As illustrated in Figs. 1 and 74 patients were excluded from the study: 27 due to other causes of elevated intracranial pressure, and 47 owing to loss to follow-up after diagnosis. The final cohort comprised 178 eligible patients, who were categorised into two groups based on whether obese or not: obese group(BMI≥28 kg/m^2^) and non-obese group(BMI<28 kg/m^2^). The overall cohort comprised 148 female patients (83.15%) and 30 male patients (16.85%). Within the non-obese group, the mean age was 37.94 ± 17.05 years with a mean BMI of 24.04 ± 2.77 kg/m², and the majority were female (64 cases, 78.05%). Among the obese group, the mean age was 34.15 ± 8.74 years with a mean BMI of 31.97 ± 3.48 kg/m², and 84 cases(87.50%) were female.

The patients in the non-obese group were significantly older than those in the obese group(*P* = 0.044) (Table 1). According to body weight classification, the non-obese group consisted of 63 overweight (76.83%) and 19 normal-weight IIH patients (23.17%), as detailed in Table 1. The mean BMI in the non-obese IIH group was significantly lower than that of the obese IIH group (24.04 ± 2.7 vs.31.97 ± 3.48 kg/m²; *P* < 0.01).

**Table 1:** Clinical features of the study cohorts on initial presentation

Sex Male	non-obese(n = 82)	Obese (n = 96)	P value
	18, 21.95%	12, 12.50%	0.093
Female	64, 78.05%	84, 87.50%	
Age, years	37.94 ± 17.05	34.15 ± 8.74	0.044
BMI, kg/m2	24.04 ± 2.77	31.97 ± 3.48	<0.01
Visual impairment	70cases(85.37%) 135 eyes	81cases(84.38%) 140 eyes	0.854
Headache	36, 43.90%	42, 43.75%	0.984
Transient visual obscuration	40, 48.78%	59, 61.46%	0.090
Tinnitus	19, 23.17%	25,26.04%	0.658
Diplopia	10, 12.20%	12, 12.50%	0.951
Radiological signs	48, 58.54%	70, 72.92%	0.043
Empty sella	36, 43.90%	62, 64.58%	0.006
DPSS	20, 24.39%	19,19.79%	0.460
TVSS	27, 32.93%	29,30.21%	0.697
BMI < 24	19, 23.17%	0	
24–28	63༌ 76.83%	0	
Risk factors OSAS	36 (43.90%)	42(43.75%)	0.984
Over-weight	63(76.83%)		
Weight gain	12(14.63%)	6(6.25%)	0.064
≥ 28	0	96, 100%	
Outcome Resolved	50, 60.98%	78, 81.25%	0.003
Improved	19, 23.17%	10,10.42%	0.022

Abbreviations: *BMI* body mass index, *DPSS* distension of the periopticsubarachnoid space, *TVSS* transverse venous sinus stenosis, OSAS obstructive sleep apnoea syndrome

According to the medical records, in the entire cohort, a total of 30 patients(36.59%) were misdiagnosed with other diseases before being diagnosed with IIH: optic neuritis in 17 cases, anterior ischemic optic neuropathy(AION) in 8 cases, and optic vasculitis in 5 cases. In the non-obese group, 18 individuals (21.95%) had been misdiagnosed, while in the obese group, 12 cases (12.5%) misdiagnose. There was no statistical difference in the misdiagnosis rate between the two groups of patients*P* = 0.093).

### Before treatments (on initial presentation)

All patients exhibited bilateral papilloedema of varying severity. Visual impairment was the most frequent presenting complaint in each cohort, with no significant difference in prevalence between the non-obese and obese groups (85.37% vs. 84.38%; *P* = 0.854; Table 1).

BCVA was measured using a standard international visual acuity chart (0.1 = 20/200, 0.4 = 20/50, 0.5 = 20/40 in Snellen chart). Within the non-obese IIH group, 70 cases patients (85.37%) demonstrated a reduction in BCVA in 135 out of 184 eyes (73.37%). Of these, 32 eyes (23.70%) showed severe decline, 26 eyes (19.26%) moderate decline, and 77 eyes(57.04%) mild decline. In contrast, patients in the obese IIH group exhibited better BCVA: 4 eyes (2.86%) presented with severe decline(*P*<0.01), while 36 eyes (25.71%) showed moderate decline (*P* = 0.200) and 100 eyes (71.43%) mild decline (*P* = 0.013) (Table 2). Humphrey visual field assessment revealed more severe defects in the non-obese IIH group. The mean visual field defect grade was 2.65 ± 1.22 in non-obese cases compared with 1.95 ± 0.94 in obese cases (*P* = 0.027). The median MFS scores were comparable between the two groups (3.55 ± 1.16 vs. 3.41 ± 0.88, *P* = 0.677), indicating similar severity of papilloedema(Table 3).

**Table 2:** Visual acuity outcomes at final follow-up

Initial BCVA<0.1	non-obese IIH (*n* = 135 eyes)	Obese (*n* = 140 eyes)	*P* value
	32 eyes, 23.70%	4eyes, 2.86%	<0.01
0.1–0.4	26 eyes,19.26%	36 eyes, 25.71%	0.200
>0.5	77 eyes,57.04%	100 eyes,71.43%	0.013
Final BCVA<0.1	11 eyes,8.15%	2eyes, 1.43%	0.009
0.1–0.4	30 eyes, 22.22%	18 eyes, 12.86%	0.041
>0.5	94 eyes, 69.63%	120 eyes, 85.71%	0.001

**Table 3:** Clinical data at initial stage (before treatment)and final follow-up

	VF Defect Grade			MFS grade			CSF OP(cmH2O)		
	Non-obese	Obese IIH	*P* value	Non-obese	Obese IIH	*P* value	Non-obese	Obese IIH	*P* value
Initial	2.65 ± 1.22	1.95 ± 0.94	0.027	3.55 ± 1.16	3.41 ± 0.88	0.677	35.01 ± 6.06	34.50 ± 5.50	0.105
Final	0.68 ± 0.67	0.46 ± 0.51	0.034	0.75 ± 0.56	0.77 ± 0.61	0.701	16.76 ± 3.06	18.60 ± 1.77	0.038

Abbreviations: *CSF* cerebrospinal fluid, *VF* visual field, *MFS* modifiedFrisén Scale.

In addition to visual impairment, the non-obese IIH group exhibited other symptoms, including transient visual obscurations in 40 cases (48.78%), headache in 36 (43.90%), tinnitus in 19 (23.17%), and diplopia in 10 (12.20%)(Table 1). The prevalence of these clinical manifestations did not differ significantly from that observed in the obese group.

## Laboratory analyses

All patients underwent routine haematological, biochemical, and coagulation testing. Further specialised serological analyses included assays for serum aquaporin-4 antibody (anti-AQP4-IgG), myelin oligodendrocyte glycoprotein antibody (MOG-IgG), double-stranded DNA antibody (anti-dsDNA), antinuclear antibody (ANA), extractable nuclear antigen (ENA), C-reactive protein (CRP), antineutrophil cytoplasmic antibody (ANCA), mitochondrial DNA (mtDNA) mutations and CSF analysis. Laboratory results were negative across all participants in both groups.

### Lumbar puncture (LP)

Lumbar puncture was performed in all patients as part of the routine diagnostic workup. ICP was confirmed via LP performed in the lateral decubitus position, with an opening cerebrospinal fluid pressure (CSF-OP) ≥ 25 cm H₂O considered diagnostic. LP opening pressure was measured using a standard manometer with the patient in a relaxed, lateral decubitus position with the neck and legs extended. LP demonstrated a CSF-OP exceeding 25 cmH₂O in all patients. Although the mean CSF-OP was markedly elevated in both groups, no statistically significant difference was observed between them (35.01 ± 6.06 cmH₂O vs. 34.50 ± 5.50 cmH₂O, *P* = 0.105; Table 3).

## Neuroimaging

A total of 155 patients underwent cranial magnetic resonance imaging(MRI) and orbital MRI; 136 patients completed magnetic resonance venography (MRV) examination; 64 patients underwent CTV examination; and 12 patients underwent digital subtraction angiography (DSA).

**Fig. 2:**
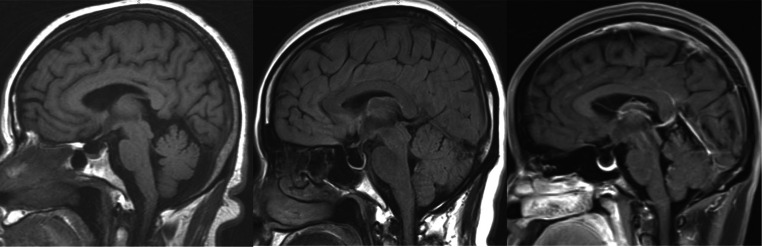
Empty sella on sagittal fat-suppressed T1WI, T2WI FLAIR and Gd-enhanced T1W1

**Fig. 3:**
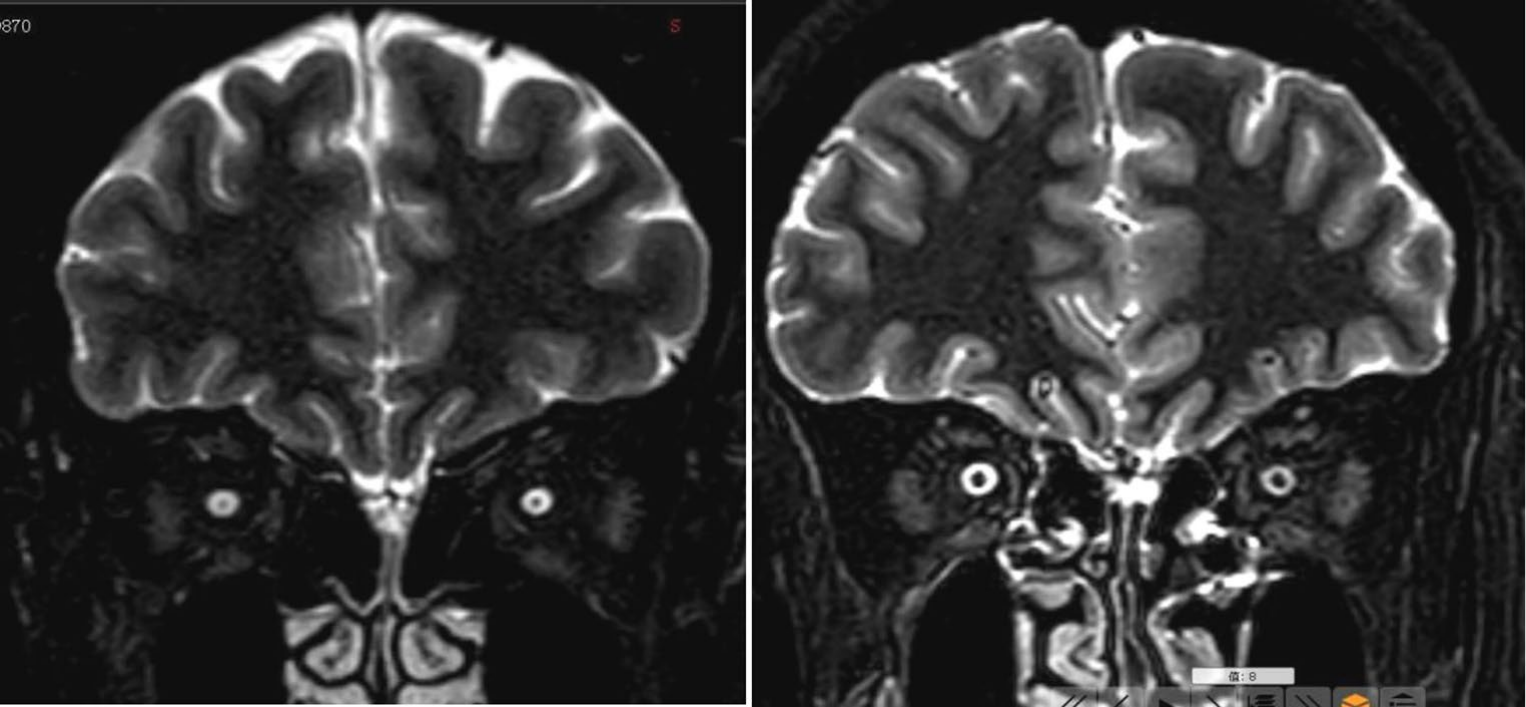
Distension of the perioptic subarachnoid(DPSS) on the coronal T2- weighted orbital MRI. Left: DPSS on both side. Rgiht: DPSS on right side

Neuroimaing examinations were evaluated for the presence of the radiological signs of intracranial hypertension as defined by the Friedman criteria [7], including empty sella, distension of the perioptic subarachnoid space (DPSS), and transverse venous sinus stenosis (TVSS). In the non-obese IIH group, abnormal radiological signs were identified in 48 patients (58.54%). Among these, empty sella was observed in 36 cases (43.90%; Fig. 2), and DPSS was present in 20 patients (24.39%; Fig. 3). TVSS was demonstrated in 27 patients (32.93%) within the non-obese group (Table 1).

By comparison, the obese IIH group demonstrated a significantly higher prevalence of abnormal radiological signs relative to the non-obese group (72.92% vs. 58.54%%; *P* = 0.043). Notably, the incidence of empty sella was substantially higher in the obese group (64.58%) compared to the non-obese cohort (*P* = 0.006; Table 1).

## Risk factors

Several comorbid conditions were identified among the participants in non-obese group. Of this cohort, 71 patients (86.59%) presented with potential risk factors associated with elevated ICP. These included 63 cases(76.83%) of over-weight, 36 cases(43.90%) of obstructive sleep apnoea syndrome (OSAS), and 12 patients(14.63%) of weight gain (weight gain greater than 5% in several months). While within obese group, 51cases (53.13%) presented with similar risk factors except for obesity(*P*<0.01). These included 42 cases(43.75%) of OSAS, and 6 patients (6.25%) of weight gain (Table 1). The incidence of weight gain was higher in the non-obese group compared to the obese cohort, but no statistically significant difference was observed (14.63% vs. 6.25%, *P* = 0.064) .

### Treatment

A range of treatment modalities were utilised for the 178 patients included in this study. Medical management emphasised weight control and included acetazolamide (250 mg per tablet) at a daily dosage of 1000–1500 mg for all patients. 35 patients received analgesic medications including ibuprofen and paracetamol (acetaminophen) for the headache management. Of these, only 2 patients still required short-term oral analgesics, while headache resolved and analgesics were discontinued in the remaining patients at final follow-up. 11 individuals (6 non-obese and 5 obese cases) underwent surgical intervention, consisting of venous sinus stenting in 7 cases and lumboperitoneal shunt placement in 4 cases.

### At final follow-up

The mean duration of final follow-up was 15.02 ± 2.82 months. At final follow-up, symptoms had resolved completely in 50 patients (60.98%) within the non-obese group, while a further 19 cases (23.17%) exhibited symptomatic improvement. The rate of complete symptom resolution was significantly higher in the obese IIH group compared to the non-obese group (81.25% vs. 60.98%; *P* = 0.003) (Table 1).

Only 44 IIH patients’ body weight information were obtained from clinical records, including 12 cases of the non-obese group and 32 cases of the obese group. Among these patients, only 15 patients achieved a weight loss of more than 5% (6 non-obese and 9 obese), while others showed no obvious changes in body weight compared to the initial stages of the disease. In addition, during the follow-up period, all patients had no recurrence.

Compared with the non-obese group, a significantly greater proportion of patients in the obese IIH group exhibited better visual outcomes at final follow-up. 2 eyes of severe visual decline were observed in the obese group(1.43% vs.8.15% in non-obese group, *P* = 0.009). Moderate decline was present in 18 eyes (12.86% vs. 22.22% ; *P* = 0.041), and mild decline in 120 eyes (85.71% vs. 69.63%; *P* = 0.001) (Table 2). Furthermore, 85.71% of eyes in the obese group achieved a BCVA above 0.5, compared with 69.63% in the non-obese group.

In the non-obese group, the mean MFS score decreased from 3.55 ± 1.16 to 0.75 ± 0.56 (Table 3). In the obese group, the mean MFS score decreased from 3.41 ± 0.88 to 0.77 ± 0.61. At the final follow-up, there was no significant difference in MFS scores between the two groups (*P* = 0.701). The mean visual field defect grade in the non-obese group improved from 2.65 ± 1.22 to 0.68 ± 0.67, while in the obese group it improved from 1.95 ± 0.94 to 0.46 ± 0.51. Despite these improvements, visual field defects remained significantly more severe in the non-obese group (*P* = 0.034). CSF-OP decreased to 16.76 ± 3.06 cmH₂O in the non-obese group and to 18.60 ± 1.77cmH₂O in the obese group. The non-obese group showed significantly lower CSF-OP values compared to the obese group at final follow-up (*P* = 0.038).

### Statistical analysis of factors affecting visual function outcomes

Univariate analysis showed that BMI, disease duration, VF grading, and patient group were significantly associated with poor BCVA (< 0.1) outcome (*P*<0.05). BCVA before treatment and imaging findings showed marginally significant associations (*P*<0.1, Table 4).

**Table 4:** Univariate analysis of factors associated with BCVA Outcome

Variable	Good Outcome (*n* = 171)	Poor Outcome (*n* = 7)	*P*-value
Age (years, xˉ±s)	36.10 ± 10.50	39.71 ± 9.20	0.362
BMI (kg/m2, xˉ±s)	27.80 ± 3.70	24.90 ± 2.60	0.042
ICP (cmH2O, xˉ±s)	34.67 ± 5.41	35.07 ± 6.95	0.821
MFS (xˉ±s)	3.50 ± 1.00	3.10 ± 1.10	0.274
Disease duration (months, xˉ±s)	5.40 ± 6.10	16.30 ± 11.70	<0.001
VFgrading (xˉ±s)	2.20 ± 1.20	3.10 ± 1.10	0.043
Gender [n (%)]			0.753
Male	29 (16.96)	1 (14.29)	
Female	142 (83.04)	6 (85.71)	
Group [n (%)]			0.035
Non-obese	76 (44.44)	6 (85.71)	
Obese	95 (55.56)	1 (14.29)	
BCVA before treatment [n (%)]			0.065
Good	154 (90.06)	6 (85.71)	
Poor	17 (9.94)	1 (14.29)	
Imaging [n (%)]			0.072
Normal	59 (34.50)	1 (14.29)	
Abnormal	112 (65.50)	6 (85.71)	

*BMI* body mass index, *ICP* intracranial pressure, *MFS* modified Frisén Scale, *VF* visual field, *BCVA* best corrected visual acuity

Multivariate logistic regression revealed that non-obese group (OR = 9.16, 95%CI: 1.09–77.18, *P* = 0.041) and longer disease duration (OR = 1.14, 95%CI: 1.05–1.24, *P* = 0.002) were independent risk factors for poor BCVA outcome. Poor BCVA before treatment showed a marginally significant trend toward poor prognosis (*P* = 0.089). The prediction model had an AUC of 0.926, demonstrating good discriminative performance for identifying patients at risk of unfavorable BCVA outcome (Table 5).

**Table 5:** Multivariate Logistic Regression Analysis of Factors for BCVA Outcome

Variable	β	*P*-value	OR	95%CI
Group (non-obese = 1)	2.214	0.041	9.16	1.09–77.18
Disease duration	0.128	0.002	1.14	1.05–1.24
Initial BCVA (poor = 1)	1.613	0.089	5.02	0.82–30.77
Constant	-5.726	<0.001	—	—

*BCVA* best corrected visual acuity

## Discussion

IIH is a rare complex neurological disorder that primarily affects obese women of reproductive age. Non-obese cases with IIH is encountered less frequently in clinical practice, which often results in misdiagnosis or delayed treatment and may consequently compromise patient outcomes. We found that in the non-obese group, the misdiagnosis rate was 21.95% (18 individuals) before being definitively diagnosed with IIH, much higher than that in the obese group (12.5%,12 cases), although there was no statistical difference in the misdiagnosis rate between the two groups of patients(*P* = 0.093) .

In this single-centre retrospective analysis, adult non-obese patients with IIH demonstrated several distinct clinical characteristics. Compared to those obese cases with IIH, they exhibited much older (mean age 37.94 ± 17.05 years), more severe visual impairment and a lower prevalence of abnormal neuroimaging features indicative of intracranial hypertension. Furthermore, a considerable proportion(86.59%) of these patients presented with comorbid conditions associated with elevated intracranial pressure.

The most frequently reported presenting symptoms of IIH, as established in the literature, include headache (76–94%), visual disturbances (68–72%) [3]. In the present study, the majority of non-obese IIH cases presented with visual acuity decline (85.37%), followed by transient visual obscuration (48.78%), headache (43.90%). It is noteworthy that headache was not the most common symptom in the non-obese IIH group. Bruce et al. [11] reported that older patients with atypical IIH(normal BMI and aged 50 years or older) tend to exhibit visual changes more frequently and headaches less often, which is consistent with the findings of this study.

When IIH is suspected, neuroradiological evaluation should be performed, particularly including fat-suppressed gadolinium-enhanced MRI of the brain and orbits [3]. In instances where other cranial nerve involvement or comorbid pathologies are present, a thorough differential diagnosis is warranted. CTV or MRV is essential to exclude cerebral venous sinus thrombosis within 24 h [3]. In the present study, radiological signs suggestive of elevated ICP were observed in 48 cases (58.54%) in the non-obese group and 70 cases (72.92%) in the obese group. These imaging features play an important role in diagnosis of intracranial hypertension. However, the prevalence of abnormal radiological signs was significantly higher in obese cases compared to non-obese cases. Notably, the incidence of empty sella was markedly elevated in the obese group (64.58%) relative to the non-obese group. The higher rate of negative imaging findings in non-obese cases may contribute to diagnostic uncertainty and inappropriate management, rendering the accurate diagnosis more challenging and necessitating heightened clinical vigilance. The higher misdiagnosis rate among non obese patientsalso confirmed the above viewpoint.

In relation to initial symptoms and treatment outcomes, Bruce et al. [11] reported no significant differences between atypical and typical IIH cases. In contrast, the present study observed that non-obese patients with IIH exhibited significantly poorer visual outcomes compared to those in obese group.This study investigated the risk factors associated with poor BCVA outcome in patients with IIH using univariate analysis and multivariate logistic regression. The key finding was that non-obese group and longer disease duration before treatment were independent risk factors for unfavorable BCVA outcome. In the present study, non-obese patients had a nearly 9-fold higher risk of poor visual recovery compared with obese patients. An increasing number of studies have highlighted that non-obese patients represent a distinct clinical subgroup with different clinical features and potentially worse visual prognosis [12, 13]. Our results are consistent with these observations, suggesting that non-obese IIH patients may experience delayed diagnosis, more atypical presentation, or more severe optic nerve damage. Clinically, these findings indicate that non-obese patients warrant closer ophthalmic monitoring and more aggressive intervention to avoid irreversible visual loss [14, 15]. Longer disease duration was also identified as an independent risk factor, with each additional month associated with a 14% increase in the risk of poor BCVA outcome. This finding reinforces the critical role of early diagnosis and timely treatment in IIH [16, 17]. Prolonged intracranial hypertension leads to cumulative and irreversible injury to the optic nerve head, resulting in poor visual recovery even after adequate treatment [18, 19]. Therefore, shortening the time from symptom onset to diagnosis and intervention is essential to improve visual outcomes in IIH patients. In addition, poor BCVA before treatment showed a marginally significant association with poor final outcome, suggesting that initial visual impairment may serve as a practical prognostic marker. Patients with reduced visual acuity at presentation should be regarded as high-risk individuals and managed intensively [20, 21]. The present logistic model demonstrated excellent predictive performance, indicating that a combination of group, disease duration, and BCVA before treatment can effectively stratify patients at high risk of unfavorable BCVA outcome. This simple and clinically applicable model may help clinicians optimize individualized treatment strategies.

Although the exact aetiology of IIH remains unclear, obesity, over-weight, anaemia, and OSAS are widely recognised as risk factors [22–24]. The high proportion of female patients (83.15%) and the elevated rate of overweight individuals(76.83% in non-obese group) observed in our study are consistent with previously reported demographic characteristics in other populations. In a separate study, it was reported that 9% of IIH cases occurred in male patients [25], which is considerably lower than the proportion of 16.85% identified in the present cohort. 88% of participants had a BMI ≥ 30 kg/m2 in Idiopathic Intracranial Hypertension Treatment trial [26]. Even obesity was defined using the criterion of 28 kg/m^2^ in our study showed an obesity prevalence of 53.93%(96/178), which is still lower than modern studies from Europe and North America. The obesity rate was surprisingly low in Asians with IIH: 6% in India, 33% in a Taiwanese study, and 45.8% in Korea. Our study better represents typical IIH in Asians [27–29]. This discrepancy may be attributable to dietary structure, racial and ethnic differences. Multicentre studies focusing on Chinese populations may yield more representative data.

Obesity and recent weight gain are established as major risk factors for IIH, showing strong associations with both disease onset and recurrence. Although the precise mechanism by which obesity contributes to IIH remains incompletely understood, current evidence indicates that central obesity may elevate intra-abdominal and pleural pressures, potentially impairing cerebral venous return [30, 31]. This pathway is thought to play a significant role in both the development and recurrence of IIH.

OSAS is closely associated with elevated ICP [32]. The proposed mechanisms underlying this association include, first, hypoxia and hypercapnia causing cerebral vasodilation and increased cerebral blood flow, which subsequently raises arterial and central venous pressure. Second, Obesity and high BMI have been associated with OSA since central obesity can elevate abdominal pressure and thereby impair cerebral venous return. Third, jugular vein resistance may be increased by factors such as neck obesity, upper airway constriction, chronic oral ventilation, and forward head posture. OSA has been reported to occur between 4% and 60% of IIH patients [25, 33]. Studies have shown that in patients with OSA, ICP was was increased as much as 9 cmH2O during apnea episodes [34, 35]. Some cases with OSA have been described who also diagnosed with IIH at presentation, proposing a possible link between the two conditions [25, 33]. Nevertheless, the role of OSAS as a risk factor for IIH remains debated. A study within the Idiopathic Intracranial Hypertension Treatment Trial (*IIH: WT*), which included 46 female IIH patients of whom 19 had OSAS, found no significant correlation between changes in the apnea-hypopnea index (AHI) and reductions in ICP [36].

Regular follow up is essential to for evaluation of reduction in CSF-OP and progress in weight loss, except for medical therapy [1]. Although the therapeutic impact of weight loss in IIH is clear, the degree of weight loss and optimal strategy necessary to induce remission is not established and may be patient-specific. Guidelines suggest realistic targets of 1–2 kg weight loss per month, with a target of losing 15–20% of body mass over 6–12 months [37]. Lifestyle modifications aimed at reducing body weight by 5–10% have demonstrated efficacy in alleviating symptoms and improving visual outcomes [38]. The biggest challenge, however, was weight loss for our cohort, most of the obese patients did not get referred to formal weight loss programmes, and the median BMI hardly shifted at one year follow-up. Maintenance of weight loss is also a challenge, with patients on average regaining one-third to one-half of the weight that was lost at one year and returning to their original weight within 5 years [39]. Among the patients enrolled in this study, only 15 cases achieved a weight loss of more than 5% (6 non-obese and 9 obese), while others showed no obvious changes in body weight compared to the initial stages of the disease.

## Conclusion

The aetiology of IIH is multifactorial and remains incompletely understood. Timely and comprehensive clinical assessment is essential for identifying potential risk factors associated with elevated ICP. Non-obese patients with IIH tend to exhibit more severe visual impairment. Non-obese status and longer disease duration are independent predictors of poor BCVA outcome in IIH patients. Early diagnosis, prompt intervention, and intensive follow-up are particularly critical for non-obese patients and those with a prolonged clinical course.

## Limitations

Several limitations of this study should be acknowledged. First, this was a single-centre, retrospective observational investigation with a limited sample size. Consequently, the statistical power to compare clinical characteristics between obese and non-obese cases was constrained. Second, the median follow-up duration was 15.02 months; a longer observation period would be required to evaluate long-term outcomes more comprehensively. Future prospective, multi-center studies with larger samples are needed to validate these findings.

## Data Availability

All data generated or analysed during this study are included in this published article.

## Abbreviations

Idiopathic Intracranial Hypertension: (IIH)
Obstructive sleep apnea syndrome: (OSAS)
Cerebrospinal fluid: (CSF)
Intracranial pressure: (ICP)
Central nervous system: (CNS)
Best corrected visual acuity: (BCVA)
Modified Frisen Scale: (MFS)
Serum aquaporin-4 antibody: (anti-AQP4-IgG)
Myelin oligodendrocyte: (MOG-IgG)
glycoprotein antibody: (MOG-IgG)
Double-stranded DNA: (dsDNA)
Antinuclear antibody: (ANA)
Extractable nuclear antigen: (ENA)
C-reactive protein: (CRP)
Antineutrophil cytoplasmic antibodies: (ANCA)
Mitochondrial DNA: (mtDNA)
Open pressure: (OP)
Lumbar puncture: (LP)
Magnetic resonance venography: (MRV)
Three-dimensional phase-contrast: (3D PC)
Transverse sinus stenosis: (TVSS)
Magnetic resonance imaging: (MRI)
Iron deficiency anemia: (IDA)
Cerebral venous thrombosis: (CVT)
